# Magnetic Forces by Permanent Magnets to Manipulate Magnetoresponsive Particles in Drug-Targeting Applications

**DOI:** 10.3390/mi13111818

**Published:** 2022-10-25

**Authors:** Sandor I. Bernad, Elena Bernad

**Affiliations:** 1 Romanian Academy-Timisoara Branch, Centre for Fundamental and Advanced Technical Research, Mihai Viteazul Str. 24, RO-300223 Timisoara, Romania; 2 Research Center for Engineering of Systems with Complex Fluids, Politehnica University Timisoara, Mihai Viteazul Str. 1, 300222 Timisoara, Romania; 3 Department of Obstetrics and Gynaecology, University of Medicine and Pharmacy “Victor Babes” Timisoara, P-ta Eftimie Murgu 2, RO-300041 Timisoara, Romania

**Keywords:** magnetic particle, permanent magnet, magnetic force, drug targeting

## Abstract

This study presents preliminary computational and experimental findings on two alternative permanent magnet configurations helpful for magnetic drug administration in vivo. A numerical simulation and a direct experimental measurement of the magnetic induction on the magnet system’s surface were used to map the magnetic field. In addition, the ferrite-type (grade Y35) and permanent neodymium magnets (grade N52) to produce powerful magnetic forces were also examined analytically and quantitatively. Ansys-Maxwell software and Finite Element Method Magnetism (FEMM) version 4.2 were used for all numerical computations in the current investigation. For both magnets, the generated magnetic fields were comparatively studied for targeting Fe particles having a diameter of 6 μm. The following findings were drawn from the present investigation: (i) the particle deposition on the vessel wall is greatly influenced by the intensity of the magnetic field, the magnet type, the magnet size, and the magnetic characteristics of the micro-sized magnetic particles (MSMPs); (ii) ferrite-type magnets might be employed to deliver magnetoresponsive particles to a target location, even if they are less powerful than neodymium magnets; and (iii) the results from the Computational Fluid Dynamics( CFD) models agree well with the measured magnetic field induction, magnetic field strength, and their fluctuation with the distance from the magnet surface.

## 1. Introduction

Any targeting strategy’s objective, regardless of the method, is to increase therapeutic effectiveness while lowering the off-target toxicity or to achieve selectivity. Magnetic targeting is a desirable, non-invasive method of selectively accumulating magnetic nanoparticles (MNPs) in various organs or site-specific lesions. Magnetic micro- and nanoparticles are also commonly used for medicinal and diagnostic purposes [1].

With a better understanding of the physiological processes involved in the magnetic targeting of an MNP to various types of vascular disease, these therapeutic results may be improved. The standard procedure for magnetic targeting entails administering MNPs after subjecting the target site to an external magnetic field. After administration, the blood flow must first passively transport nanoparticles to the lesion. Once an MNP has been delivered into the vasculature lesion, its retention is determined by the interaction between the magnetic and hydrodynamic drag forces and the vascular wall environment [1,2].

There is still much to learn about the procedure to enable clinical translation, even though both in vitro and in vivo research has clarified the aspects affecting the magnetic targeting of MNPs. It has been said that medical doctors may not use magnetic targeting as much as they can because they do not understand how it works [3].

Simplified in vitro models frequently need insights into the different elements driving drug delivery processes because physiological systems are inherently complicated. For example, in vitro simulations using tubes of various sizes and designs subject to a magnetic field gradient are frequently used to achieve the magnetic targeting of MNPs, as previously reported [4,5,6,7,8]. MNP retention can be measured in vitro to provide an estimate of what might happen in vivo. Thus, it is feasible to investigate the effects of several factors on the efficiency of magnetic targeting, including magnet configuration, separation from the magnetic pole, flow velocity, particle size, and particle surface properties.

Additionally, external magnets should be used to decrease off-target adverse effects and maximize the effectiveness of drug delivery to the target tissue to optimize magnetic drug targeting (MDT) as a minimally invasive drug delivery approach. To understand how magnetic systems work, it is essential to know how the motion of superparamagnetic particles in a flow is affected by the force of a magnetic field that changes in space.

Currently, delivering a strong enough magnetic force to resist the shear force of the blood flow under the human body magnetic field and concentrating magnetic medications alone at a specific target site are the primary problems to be addressed for transferring fundamental research into clinical application in MDT Improving the magnetic characteristics of carriers and optimizing the magnet system are the solutions to the issues. The progress in this area has been evaluated [9,10,11,12,13,14]. Most research has been focused on making magnetic carriers with coatings that can be used for more than one thing and new ways to make them.

Understanding the kinetics of particle capture and accumulation in magnetic drug-targeting applications can have a significant impact on the design and optimization of magnetic assemblies [15,16]. The optimum technique to deliver the treatment to the target volume is frequently not considered in simulation studies. This is because magnet optimization is usually judged by the total magnetic force generated over an area of interest [17,18,19]. Less emphasis has been paid to efforts to enhance carrier transport mechanisms and magnet systems, which control the motion of magnetic particles. When making the MDT, it is essential to consider the magnetic field strength and shape of the magnet system [20,21,22,23,24,25,26].

## 2. Methods

Quantifying MNP retention in vitro allows for an estimation of what would occur in vivo. In this way, it is possible to study how characteristics (such as magnet configuration, flow velocity, particle surface features, distance from the magnetic pole, and particle size) affect the effectiveness of magnetic targeting. The straightforward tube model, however, would appear unlikely to provide an adequate portrayal of what happens in in vitro models, given the intricacy of magnetic targeting in vivo.

According to several studies, MDT is best used on objects near the magnet tip’s surface. The following intrinsic issues restrict the use of MDT applications in clinical settings:

The typical magnet system’s applied magnetics force is insufficient to effectively treat the tiny particles and fast blood flow [27]. The consensus is that a substantially larger magnetic field gradient would be needed to keep magnetic nanoparticles in large arteries under safe magnetic fields [28]. The efficiency of MDT will be reduced because magnetic particles will gather not only in the intended area but also in the areas between the magnet and the target where the fields and gradients are more vital. This argument was demonstrated in Grief and Richardson’s study [29].

Typically, a superconducting magnet, an electromagnet, or a combination of permanent magnets can all be used to create a magnetic field.

Using an appropriate configuration of NdFeB permanent magnets, in vivo tests of magnetic drug delivery and targeting a particular organ have been carried out in various research [30,31].

Therefore, this research aims to give an initial experimental investigation on two different permanent magnet configurations helpful for magnetic drug delivery in vivo. The goal is to increase the magnetic driving force between one and three centimeters from the magnet surface (i.e., a value comparable to the typical distance of an artery vessel from the skin surface). The magnet configurations were created for in vivo tests in which magnets would be positioned at a distance from the vessel wall to facilitate the concentration of the magnetic particles (MPs) at a particular location of the artery bifurcation.

### 2.1. Governing Equations

Several forces, including hydrodynamic drag forces produced by blood flow, gravity, magnetic buoyancy, and inertial forces, impinge on the magnetic particles during the magnetic targeting process. For nanoparticles, buoyancy, gravitational, and inertial forces are regarded as inconsequential because they are so tiny [32]. A lift force perpendicular to the flow direction is experienced by particles traveling in the direction of increasing velocity. This force was discovered insignificant and inferior to the drag and magnetic forces [33]. Therefore, the hydrodynamic and magnetic forces are the only two factors remaining to consider when aiming particles.

### 2.2. Hydrodynamic Force

A ferromagnetic particle experiences hydrodynamic forces due to the fluid’s and the particle’s different speeds (FMP).

The Navier–Stokes equations describe the fluid velocity in the tube flow. The velocity is determined from the parabolic equation [18,19] under the assumption that there is full laminar flow in the cross-section of a cylindrical tube.

(1) $$V_{x}=\frac{2\vec{V}}{R^{2}}\left(R^{2}-y^{2}\right),$$

*V* is the mean flow velocity, *V_x_* is the velocity *x*-component, *R* is the tube radius, and *y* is the distance from the tube center line. The dimensionless Reynolds number controls the relationship between a flow’s inertial and viscous forces.

(2) $$Re=\frac{2\rho \vec{V}R}{\mu },$$

*μ* is the fluid’s dynamic viscosity, and *ρ* is the fluid’s density, respectively. For example, spherical suspended particles follow Stokes’ rule in the case of low Reynolds number flow, taking the form [32,33].

(3) $${\vec{F}}_{d}=6\eta \pi R_{p}\left({\vec{V}}_{f}-{\vec{V}}_{p}\right),$$

where *R_p_* is the particle radius, *η* is the fluid viscosity, ${\vec{V}}_{f}$ is the fluid velocity, and ${\vec{V}}_{p}$ is the particle velocity [19].

### 2.3. Magnetic Force

When a magnetic field gradient ∇H acts on a particle with magnetic moment m, the magnetic force experienced by that particle may be represented [34] as

(4) $$F_{mag}=0.5\mu_{0}\chi V_{p}\nabla H^{2},$$

where:

*F_mag_*—magnetophoretic force;

*μ*_0_—is the magnetic permeability of vacuum (*μ*_0_ = 4π × 10^−7^ N/A^2^);

*χ*—is the magnetic susceptibility [-]

*V_p_*—is the volume of the magnetic particle (*V_p_* = $\frac{4}{3}\pi R_{p}^{3}$), *R_p_*—magnetic particle radius.

Practically, the magnetic moment obtained by the isolated particle surrounded by the fluid and put in an external magnetic field is described by Equation (4).

## 3. Materials

### 3.1. Problem Description

**Figure 1:**
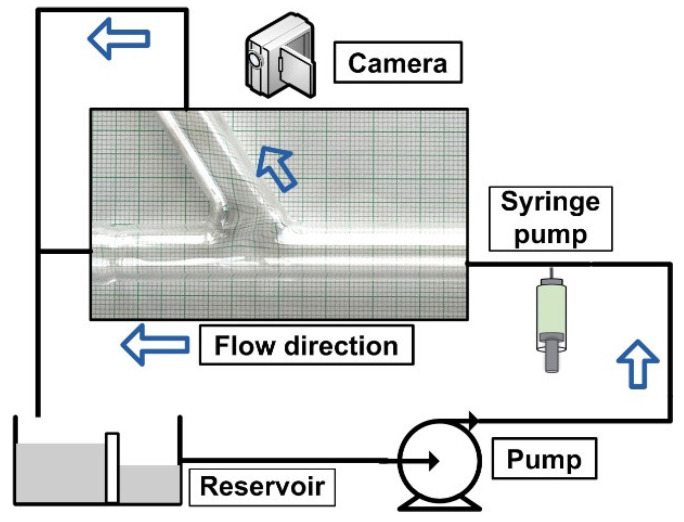
Experimental setup design.

Figure 1 shows the design of the experimental setup. The primary objective is to use an external magnetic field to attract the drug-carrying magnetic particles to the vessel’s surface. The particles were evenly dispersed throughout the vessel after injection at 10 × D (D = 8 mm, vessel diameter) from the target location. Two permanent magnet types (neodymium and ferrite magnet) produced the magnetic field. The magnetic field intensity may change by adjusting the distance between the magnet’s center and the vessel’s axis. The impacts of several parameters, including permanent magnet type, magnetic field strength, and produced magnetic force, are examined to emphasize the significance of these factors for MDT.

### 3.2. Magnetic Particle

Multi-domain soft Fe particles (Carl Roth GmbH, Karlsruhe, Germany) with a density of ρ =7.86 g/cm^3^ and size of 4–6 μm were utilized in this investigation to mimic the magnetic carrier (same as in our previous study [5]). As a result, the magnetic field needs of the experimental setup are significantly reduced by their magnetic moments, which are approximately 10^6^ ÷ 10^7^ times larger than those of typical drug-carrying iron oxide nanocomposites.

The size of the utilized particles is significantly greater than that of the drug delivery particles [35,36]. The practical selection of this kind of magnetic particle, which has a large diameter, was influenced by the findings of Pislaru et al. [37], who investigated the endothelialization of 8 mm diameter synthetic vascular grafts (commercial Dacron grafts) using coated iron oxide particles with a diameter of 0.9 mm. However, because the size of this magnetic particle is like that of a red blood cell (7.5–8.7 μm), we chose to employ it.

**Figure 2:**
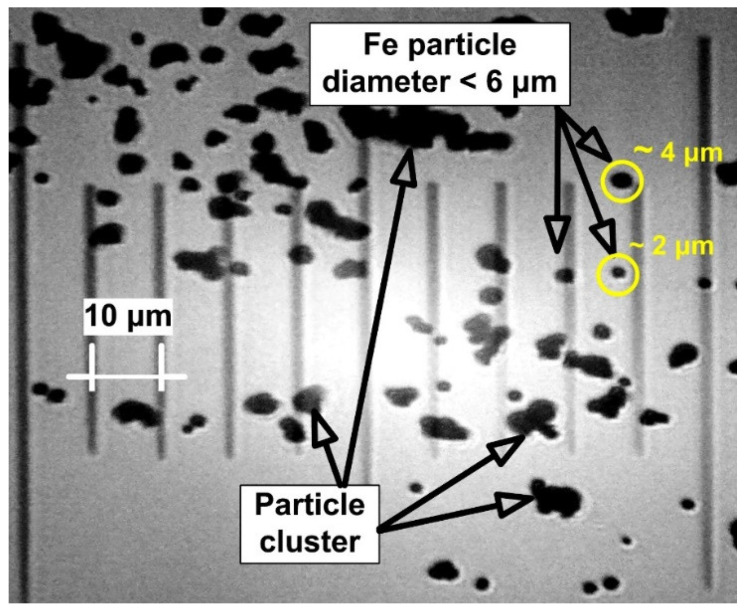
Morphology of the 4–6 μm Fe powder as seen in a microscopic picture.

A microscopic picture in Figure 2 validated the physical shape of the applied Fe particles. Therefore, the Fe particle characteristics are presented in Table 1.

The image demonstrates the presence of the particle cluster as well as variations in the particle size distribution from the given mean size.

**Figure 3:**
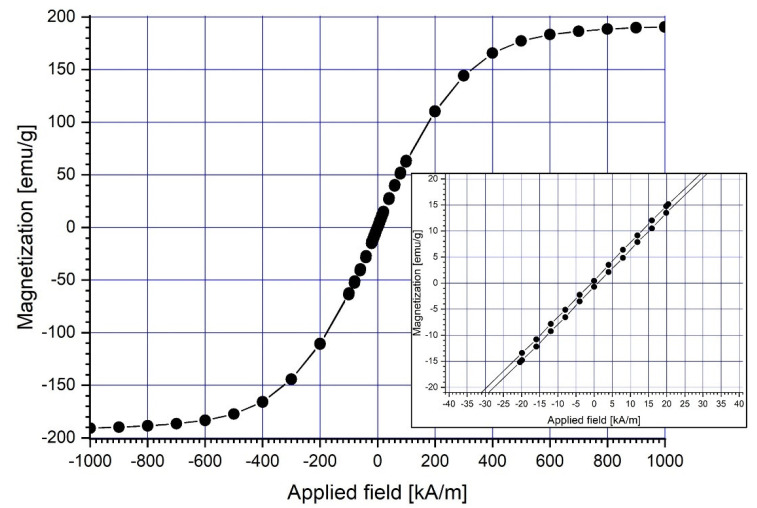
Magnetization curve of the micro-sized (4–6 μm) Fe particles.

At ambient temperature (22 °C), a vibrating sample magnetometer (VSM 880-ADE Technologies, Pensacola, FL, USA) with a field range of 0 to 950 kA/m was used to assess the magnetic characteristics of the magnetic carrier (Fe particles) (Figure 3). Table 2 displays the overall findings of the Fe magnetic characteristics.

In the picture, the hysteresis loops are smooth, and there is no hysteresis. This means that the magnetic material is soft and has coercive force, and the residual magnetization is close to zero.

### 3.3. B-Field Experimental Measurement

A Tesla meter measured the real B-field strength along the magnet’s central axis at various distances (Model 5080, F.W. Bell Gaussmeter, Milwaukie, OR). The device, therefore, measures the axial component of the induction field vector. A homemade positioner system was used to position the Hall probe precisely. The positioning setup’s measurement error along the vertical direction was calculated to be 0.5 mm. The analytical and computational B-fields from numerical simulation were compared to these measurements.

### 3.4. Magnets and Magnetic Field Generation

One of the most crucial aspects of permanent magnet design is estimating the magnetic flux distribution in a magnetic circuit, which may consist of air gaps, permanent magnets high-permeability conduction materials, and electrical currents. You must consider several intricate aspects to obtain precise information regarding magnetic fields. However, by making a few oversimplified assumptions, you may achieve immediate results.

**Figure 4:**
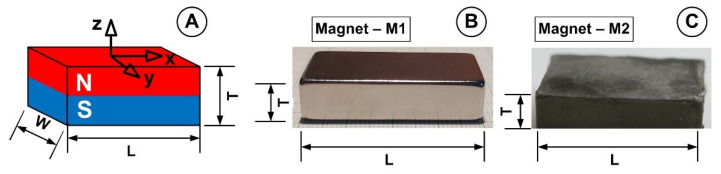
Permanent magnets used in this study. (**A**) Dimensions, axis association, and polarization direction. (**B**) Magnet M1—Neodymium-type magnet with grade N52. (**C**) Magnet M2—ferrite-type magnet with grade Y35.

Permanent magnets were used to produce the magnetic field that was applied. Neodymium-type magnets and ferrite-type magnets, two different rectangular permanent magnets, were studied in this paper (Figure 4). Table 3 and Table 4 describe the size and characteristics of the employed permanent magnets according to the supplier-provided data. Both magnets have magnetization directions axially oriented. The Neodymium-type magnet was Ni-coated (Figure 4B).

A theoretical study for the magnetic field distribution around a rectangular permanent magnet (Equation (5); [38]) was used to compare the magnetic field distribution produced by the studied permanent magnets:

(5) $$B\left(z\right)=\frac{B_{r}}{\pi }\left(\tan^{-1}\frac{WL}{2z\sqrt{4z^{2}+W^{2}+L^{2}}}\tan^{-1}\frac{WL}{2\left(z+T\right)\sqrt{4{\left(z+T\right)}^{2}+W^{2}+L^{2}}}\right),$$

*W*—magnet width, *L*—magnet length, *T*—magnet thickness, *B_r_*—is the magnet residual flux density, and *z*—distance from the magnet surface (where *z* ≥ 0) on the magnet’s centerline.

Finite Element Analysis (FEA) modeling programs analyze magnetic issues and find more precise solutions. Forces and flux densities were computed using FEA models. Ansys Maxwell commercial software (Ansys Inc., Canonsburg, PA, USA) and the freeware Finite Element Method Magnetism (FEMM) version 4.2—(http://www.femm.info/wiki/HomePage, accessed on 20 August 2022) were used for numerical simulations of the two investigated permanent magnets. The static magnetic field of the permanent magnet system is analyzed and calculated by Ansys Maxwell using the finite element method. Moreover, the freeware finite element method can solve the set of Maxwell equations and obtain the value of magnetic vector potential and magnetic flux density.

In this work, we use the two different software packages for the following reasons:

(1) To have the certainty of the correctness of the numerical simulation results obtained (by comparing the results generated by the two programs);
(2) To compare the accuracy of the numerical simulation solutions with the analytical and experimental results, respectively;
(3) To identify the effectiveness (in terms of the computation time and computations cost) of the two numerical simulation programs for solving problems related to the properties of the magnetic field associated with different objects or magnetic equipment (in our case, two different permanent magnets).

### 3.5. Experimental Test Rig

Figure 4 depicts the experimental configuration used to simulate the magnetic drug targeting in the arterial bifurcation region.

**Figure 5:**
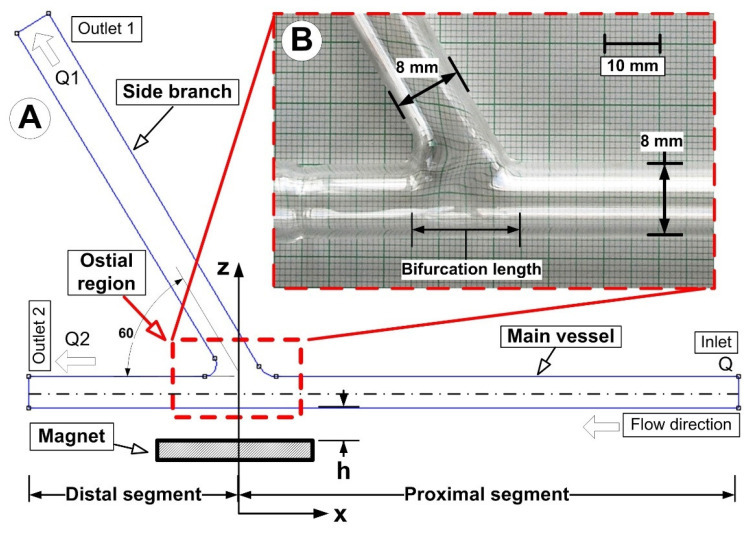
The investigated artery bifurcation model: (**A**) numerical model; (**B**) experimental model. The constant internal diameter of 8 mm was used for numerical and experimental model generation (both, for the main vessel and the branch).

The artery bifurcation model was fed with a flow of carrier fluid (a water-glycerin combination) by a computer-controlled centrifugal pump (from the German company GAMPT mbH, Merseburg, Germany) (Figure 1). The test section, made from glass tubing with a 110 mm long proximal segment and a 70 mm long distal segment, was an arterial bifurcation with a bifurcation angle of 60° (Figure 5). The main flow was adjusted to 0.12 m/s, giving a flow rate of Q = 362 mL/min corresponding to Reynolds number of Re = 281. Before the flow entered the intake portion of the bifurcation model, the magnetic particles (Fe particles) were discharged into a continuous laminar main flow for 30 s. A permanent magnet was positioned in various vertical locations in the artery bifurcation to provide the necessary magnetic field. According to Equation (6), the shear rate, *γ*, for an 8 mm tube is 15 s^−1^ (assuming laminar flow):

(6) $$\gamma =\frac{4Q}{\pi R^{3}}$$

*R* is the tube’s internal radius, and *Q* is the volume flow rate.

It is significant to note that the wall roughness in the current study is minimal because the test section is made of glass. As a result, this work did not evaluate this kind of characteristic.

### 3.6. The Carrier Fluid

This investigation utilized a fluid with the same density as blood (1055 kg/m^3^) as the working fluid (carrier fluid—CF). The glycerol-water solution in use guarantees that the rheological behavior of blood is reproduced [5,6,39]. The aqueous glycerol solutions employed as the carrier fluid were combined with iron (Fe) particles at a mass concentration of 5% (corresponding to 1 g Fe dispersed in 20 mL carrier fluid) to create the model suspension of magnetic carriers utilized in the studies.

## 4. Results

In our model, the magnetic force produced by the permanent magnets (F_mag_) and the viscous force caused by the flow were considered (F_d_). The magnetic particles in our experiment have a diameter of around 6 μm. The difference between the velocity of the particles and the flow surrounding them is minimal because the particles are inertia-free. As a result, there is little hydrodynamic contact between the particles. To represent how the MSMPs transport with the flow, three independent steps can be taken:

(1) Calculating the permanent magnets’ magnetic fields.
(2) Calculating the magnetic force of a magnetic particle.
(3) An experimental investigation of the particle deposition for both mentioned magnets.

### 4.1. Calculation of the Permanent Magnets’ Magnetic Fields

**Figure 6:**
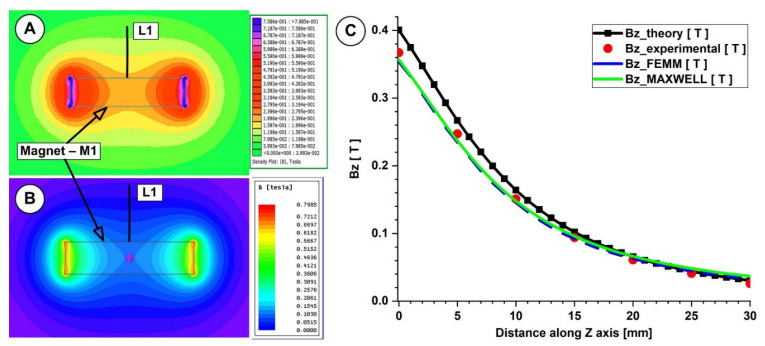
Theoretical and numerical investigations of the magnetic field generated by the magnet M1 (40×20×10 mm), type NdFeB grade N52. Numerical results: (**A**) using FEMM 4.2, (**B**) using Ansys Maxwell. (**C**) *B_z_* evolution function of the magnet surface distance. Comparison between theoretical, experimental, and numerical results.

**Figure 7:**
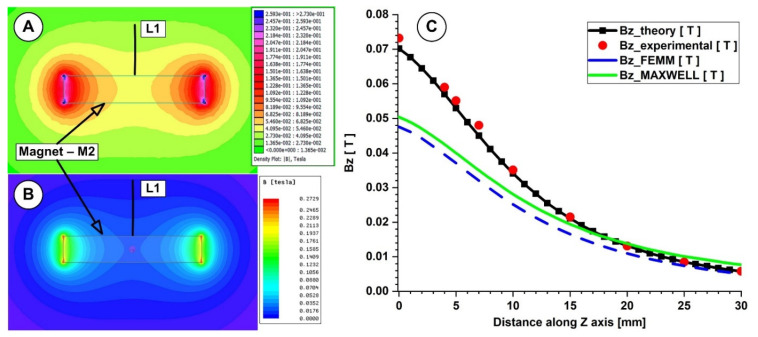
Theoretical and numerical investigations of the magnetic field generated by the magnet M2 (29×26×5 mm), type ferrite grade Y35. Numerical results: (**A**) using FEMM 4.2, (**B**) using Ansys Maxwell. (**C**) *B_z_* evolution function of the magnet surface distance. Comparison between theoretical, experimental, and numerical results.

This work compared the evolution of the magnetic field obtained from the numerical simulation, from the analytical investigation, and from the experimental measurements (Figure 6A–C and Figure 7A–C).

In both measurements, for the investigated magnets, the B-field intensities decrease exponentially with the increasing distance from the magnet’s surface. The experimentally determined peak B-field magnitude for the neodymium magnet was roughly 380 mT, but for the ferrite magnet, it was just 70 mT. The B-field magnitude ranged between 270 and 120 mT for the neodymium magnet and between 55 and 22 mT for the ferrite magnet at a distance between 5 and 15 mm from the magnet surface (the distance of interest for our experiments). Figure 6 and Figure 7 show that the experimentally measured B-field values and the corresponding analytically determined values (Table 5 and Table 6) were in good accordance. Equation (7) estimates the errors between the findings from the analytical model, experimental data, and FEA results. For the two permanent magnets under investigation, the errors are presented in Table 5 and Table 6 (Equation (7) adapted for each estimated error).

(7) $$\%\;{\mathrm{diff}\;\mathrm{equation}}_{\exp}=\left|\frac{\mathrm{Bz}\_\mathrm{equation}-\mathrm{Bz}\_\exp}{{\mathrm{Bz}}_{\mathrm{eqaution}}}\right|\times 100\%$$

As shown in Table 5, for the neodymium N52 permanent magnet, the B-field values obtained analytically agree well with the values obtained from the experimental and numerical investigations (for both used numerical software), and the percentage differences are around 10%. However, in the case of the ferrite Y35 permanent magnet (Table 6), we have a consistent difference between the numerical and analytical results. In our opinion, the difference between the theoretical and experimental values in both situations is due to the spatial position of the gaussmeter hole.

**Figure 8:**
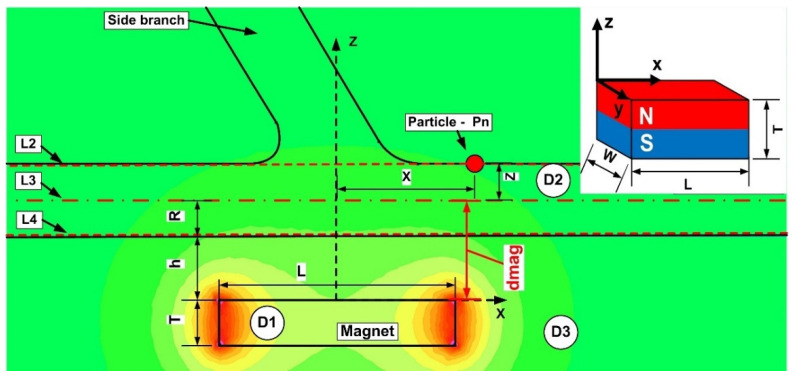
Definition of the investigation sections during numerical analysis. L1, L2, and L3 (dashed lines) depict the site of investigation of the magnetic field strength H. Figure shows the magnitude of the magnetic field generated produced by the ferrite-type magnet. The magnet is placed at h mm from the vessel’s bottom wall. This study employs three distinct domains: (**D1**)-permanent magnet; (**D2**)-carrier fluid; and (**D3**)-air.

**Figure 9:**
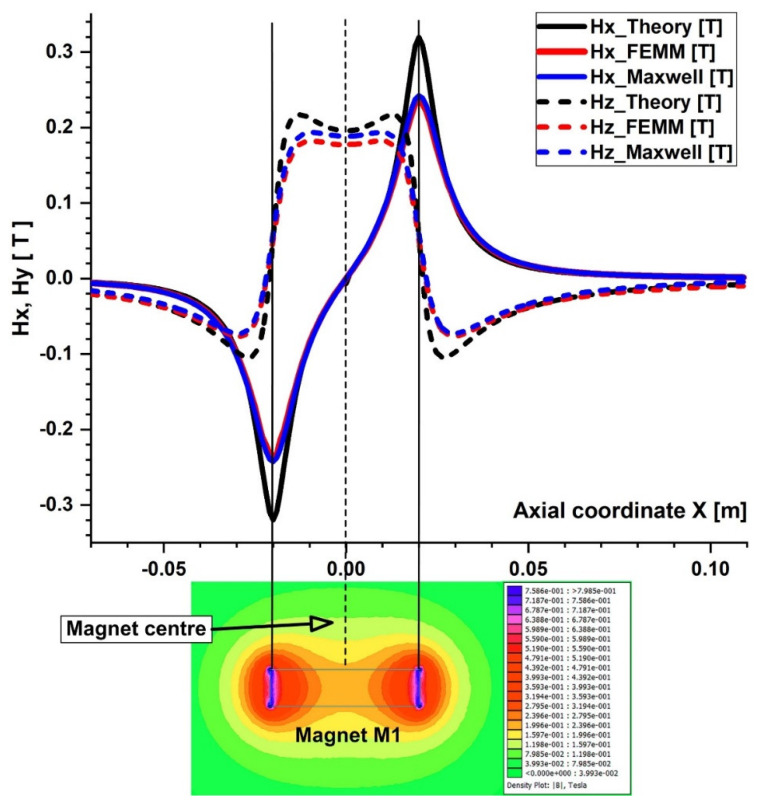
Comparison of the magnetic field components $H_{x}\;\mathrm{and}\;H_{y}$ obtained analytically, experimentally, and using FEA (FEMM and Maxwell software) along the x direction of the bottom wall of the vessel. B−field magnitude distribution for neodymium permanent magnet M1 grade N52 bottom. The distance between the vessel’s bottom wall and the magnet surface is h = 5 mm.

**Figure 10:**
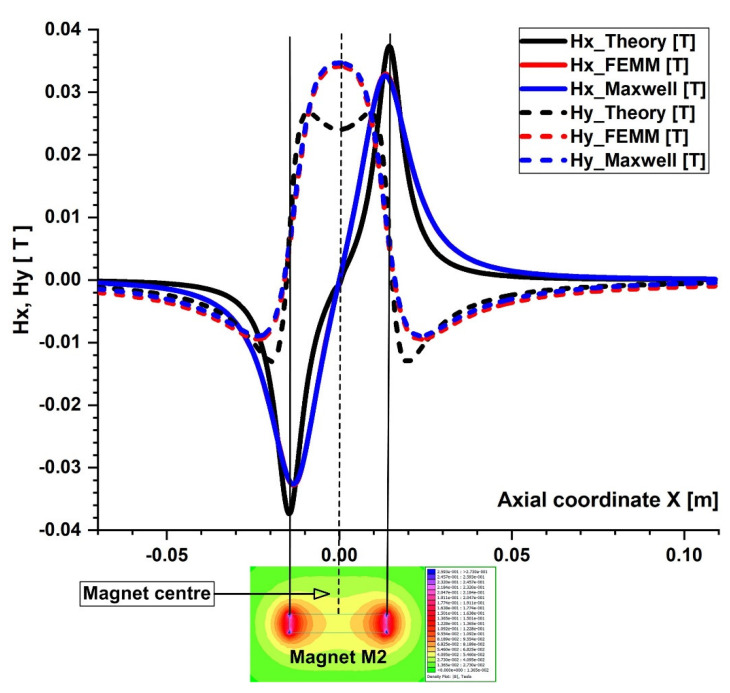
Comparison of the magnetic field components $H_{x}\;\mathrm{and}\;H_{y}$ obtained analytically, experimentally, and using FEA (FEMM and Maxwell software) along the x direction of the bottom wall of the vessel. B−field magnitude distribution for the permanent ferrite magnet (magnet M2) grade Y35 bottom. The distance between the vessel’s bottom wall and the magnet surface is h = 5 mm.

In the x-z plane, the magnetic field simulations are run. Consequently, two components are along the x and z axes in the magnetic field and the magnetic force acting on the particles (Figure 8). Figure 9 and Figure 10 depict the magnetic field components produced by the examined magnets along the vessel’s central axis (the magnets in Figure 4).

The particles are drawn toward the magnet by the gradient of the magnetic force produced by the z-component field, Hz, in the z direction. As an illustration, the magnet’s center has the maximum value of Hz = 0.355 T for magnet M1 (Figure 6). These graphs also demonstrate a substantial Hz decrease away from the magnet and toward the tube’s intake and exit. Significant edge effects can be seen in the spatial gradient of the B-field for the rectangular magnet M1, where the force component is greatest close to the corners. To determine the magnetic field strength (H) components of the associated permanent magnet, the analytical solutions of Equations (8) and (9) were confirmed numerically using FEMM, a freeware program, and Ansys-Maxwell, a commercial program (Figure 9). The x component of the field, Hx, oscillates along the vessel’s axis, and its negative peak values are close to the magnet’s margins (x = ± 20 mm from the magnet’s M1 center). Our findings agree quite a bit with those shown in the cited source [40]. Figure 10 illustrates the evolution of the magnetic field components for magnet M2 (a ferrite magnet, grade Y35), which is identical to that of magnet M1

(8) $$H_{x}\left(x,z\right)=\frac{M_{s}}{4\pi }\left[ln\left(\frac{{\left(x+\frac{L}{2}\right)}^{2}+{\left(z+d_{mag}\right)}^{2}}{{\left(x+\frac{L}{2}\right)}^{2}+{\left(z+d_{mag}+T\right)}^{2}}\right)-ln\left(\frac{{\left(x-\frac{L}{2}\right)}^{2}+{\left(z+d_{mag}\right)}^{2}}{{\left(x-\frac{L}{2}\right)}^{2}+{\left(z+d_{mag}+T\right)}^{2}}\right)\right],$$

(9) $$H_{z}\left(x,z\right)=\frac{M_{s}}{4\pi }\left[\tan^{-1}\left(\frac{T\left(x+\frac{L}{2}\right)}{{\left(x+\frac{L}{2}\right)}^{2}+{\left(z+d_{mag}+\frac{T}{2}\right)}^{2}-{\left(\frac{T}{2}\right)}^{2}}\right)-\tan^{-1}\left(\frac{T\left(x-\frac{L}{2}\right)}{{\left(x-\frac{L}{2}\right)}^{2}+{\left(z+d_{mag}+\frac{T}{2}\right)}^{2}-{\left(\frac{T}{2}\right)}^{2}}\right)\right],$$

where *d_mag_* = h + R, h—distance from magnet surface to the vessel wall, R—vessel radius, according to Figure 7.

### 4.2. Calculation of the Magnetic Force Acting on the Micro-Sized Magnetoresposive Particle

Under the following restrictive assumptions, both the analytical and numerical solution for the magnetic force generated by the external magnetic field was calculated: the flow is entirely developed, the applied magnetic field is homogeneous, and the particle Reynolds number is low.

Figure 7 presents the different domains used in this work. The magnetic flux density in these domains can be described using the connection between H and B [40].

(10) $$B=\{\begin{array}{c} \mu_{0}\mu_{r,mag}H+B_{r};\;\mathrm{for}\;\mathrm{magnet}\;\left(\mathrm{domain}\;D1\right)\\ \mu_{0}\left(H+M_{b}\left(H\right)\right);\;\mathrm{for}\;\mathrm{carrier}\;\mathrm{fluid}\;\left(\mathrm{domain}\;D2\right)\\ \mu_{0}H;\;\mathrm{for}\;\mathrm{air}\;\left(\mathrm{domain}\;D3\right) \end{array}$$

where *B* is the magnetic flux density (T), *μ_r,mag_* is the relative permeability of the permanent magnet, *H* is the magnetic field strength (A/m), and *M_b_* (*H*) is the magnetization vector of the model suspension stream (A/m) (is a function of *H*). As mentioned in the previous paragraph, we used Fe particles as the magnetic particles in this study. The magnetic properties of the Fe particle and the used carrier fluid are shown in Table 7. Also, all the parameters used in this paper for calculation are defined in Table 8.

Numerous forces work on magnetic particles in viscous environments and magnetic fields as MNPs move through the circulatory system [40], Equation (11).

(11) $$m_{eff}\frac{dv_{p}}{dt}=F_{mag}+F_{d}+F_{g}+F_{bouy},$$

where *m_eff_*—is the particle’s mass, including the added mass; $v_{p}$—is the particle total velocity; *F_mag_*—magnetophoretic force; *F_d_*—fluid drag force; *F_g_*—gravitational force; and *F_bouy_*—Buoyancy force.

The dispersion of small particles (with a diameter ≤50 nm) is greatly influenced by Brownian motion and the associated stochastic forces [41,42]. Given that we utilized Fe particles with a diameter of 4–6 μm in our experiments, Brownian motion is disregarded because the particle diameter was chosen to be more than 50 nm [34]. In the momentum equation, the magnetic field of the permanent magnet was treated as a source term. Due to the MPs’ small size, gravity was disregarded, even though its impact pales compared to the external magnetic field’s greater body force. Additionally, we consider particle inertia to be minimal.

By balancing the hydrodynamic drag and magnetic forces, we can obtain the velocity of MSMPs (*v_mag_*) in the model suspension solution, *v_mag_* = F*_mag_*/6πμ R_p_, where R_p_ denotes the radius of an MSMP.

The total velocity *v_p_* of the MPs is [43]:

(12) $$v_{p}=v_{f}+v_{mag},$$

F = nF*_mag_*, where n is the number of MPs in the model suspension, was used as the magnetic force per unit volume assumption. Equation (4) provides the magnetic force operating on each particle.

The magnetic particle velocity *v_mag_* is [44]:

(13) $$v_{mag}=\frac{\mu_{0}\chi_{Fe}R_{p}\nabla H^{2}}{9\eta_{f}},$$

The hydrodynamic forces acting on an MP are caused by variations in the velocities of the fluid and particles. Assuming a Newtonian fluid, Stokes’ equation [40] provides the drag force acting on the spherical particle as described in Equation (3).

We consider the motion in the x–z plane, and therefore, the *x* and *z* components of the fluid velocity can be expressed as:

(14) $$v_{f_{x}}=2v_{m}\left[1-{\left(\frac{z}{R}\right)}^{2}\right],$$

(15) $$v_{f_{z}}=\frac{R_{p}^{2}\left(\rho_{p}-\rho_{f}\right)g}{9\eta },$$

**Figure 11:**
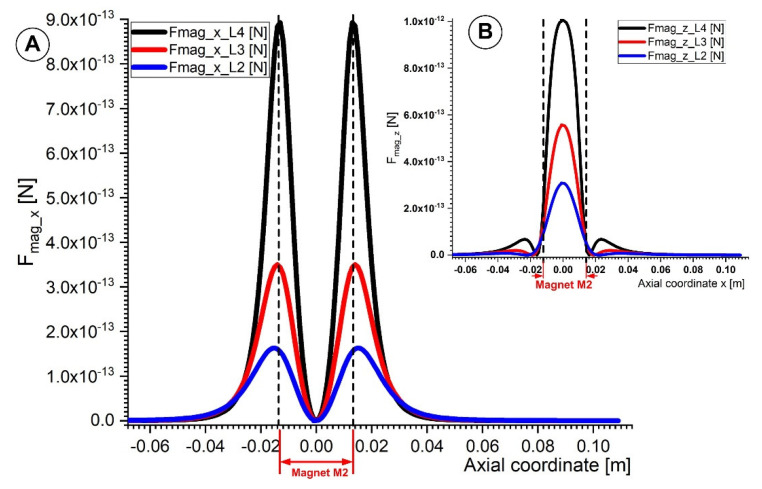
Comparison of the magnetic force components for magnet M2 along the vessel axial coordinate. (**A**) Evolution of magnetic force x and z components at various distances from the magnet surface (lines L4, L3, and L2 in Figure 8). (**B**) A comparison of the vertical magnetic force component F_mag_z_ along the lines L4, L3, and L2.

Figure 11 depicts the vertical and horizontal components of the magnetic force acting on the 6 μm diameter Fe particle. The axial component of the magnetic force F*_mag_*__x_ is most significant on the left and right sides of the magnet (Figure 11A). The magnetic particle is drawn to the magnet surface by the horizontal magnetic force. As a result, the magnetic nanoparticles move toward the magnet more quickly and away from it more slowly. On the other hand, the magnet’s center is where the vertical component of the magnetic force achieves its highest magnitude (Figure 11B). The magnetic micro-sized particles are caught and collected by this magnetic force as they travel within the vessel.

This magnetic force must be greater than the drag force to trap the flowing magnetic particles inside the artery. As demonstrated in Figure 11, the magnetic particle capture effectiveness rises with the strength of the magnetic field (in other words, by decreasing the distance between the magnet surface and the artery wall).

The axial and vertical components of the magnetic force presented in Figure 11 were calculated using Equation (4), whereas the magnetic field gradient ∇H components were calculated using Equations (8) and (9).

## 5. Discussion

As shown in Figure 11, the oscillatory movement of the micro-sized particles inside the artery is caused by the horizontal component of the magnetic field (F_mag_x_). In addition, the magnetic force’s vertical component (F_mag_z_) strength is inversely proportional to the distance from the magnets’ centers to their edges and are responsible for particle deposition.

**Figure 12:**
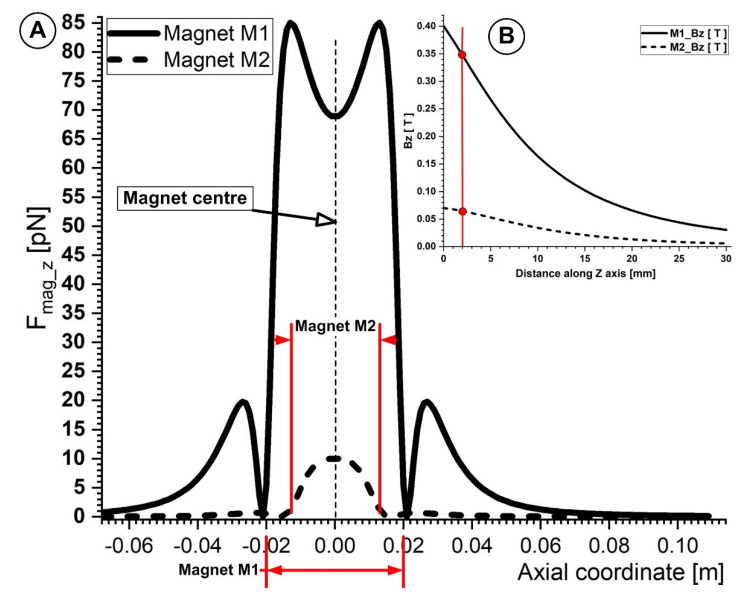
(**A**) Comparison of the magnetic force component F_mag_z_ generated by magnets M1 and M2 along the bottom wall of the vessel. The centers of both magnets are placed in the same position as the vessel bifurcation center. For both magnets that were looked at, the distance between the bottom of the vessel and the magnet surface was h = 2 mm. (**B**) The magnetic field induction B_z_ (T) produced by magnets M1 and M2 are compared along the *z*−axis as a function of the distance to the magnets’ surfaces. Due to the different magnet types (ferrite (M2) and neodymium (M1)) and magnet grades (Y35 and N52, respectively), there are significant variances between the magnetic field inductions generated by the investigated magnets.

Figure 12A,B illustrates how weaker ferrite-type magnets are compared to NdFeB magnets. Typically, the draw force of ferrite-type magnets is around one-seventh that of NdFeB magnets of the same size. Its key benefits are NdFeB’s magnetic characteristics, strong coercivity, and remanence. NdFeB-type permanent magnets may be found in strengths up to 1.48 Tesla [45] in their magnetic characteristics. This is crucial from the standpoint of targeting because this kind of magnet may be used to target magnetoresponsive particles in deeper-seated arteries throughout the body. For instance, Lubbe et al. [45] employed permanent magnets to target particles of up to a 5 cm depth during human experiments. Additionally, Goodwin [46] reported that targeting depths of up to 12 cm were discovered during animal experimentation.

To analyze and assess the ferromagnetic particle deposition inside the vessel bifurcation, an in vitro flow model was developed for this work. 

### 5.1. The Effect of Flow Rate on Particle Accumulation Evolution

**Figure 13:**
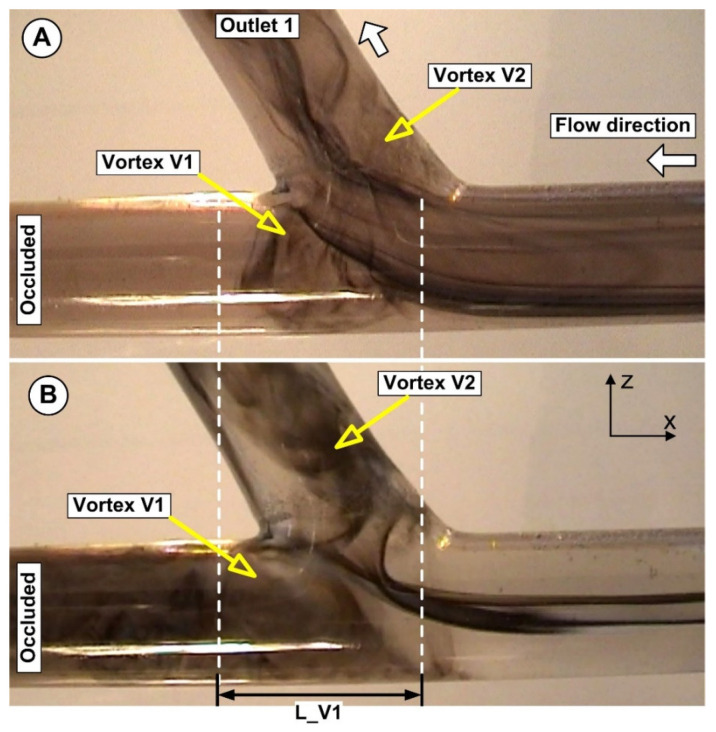
Flow field evolution in the artery bifurcation. (**A**) For a flow rate of Q1 = 362 mL/min and (**B**) for a flow rate of Q2 = 754 mL/min. The bifurcation induces the generation of two recirculation areas, one in the main artery (Vortex V1) and the other in the side branch (Vortex V2). The shape and extension of both vortices are directly related to the flow rate and the bifurcation angle.

The bifurcation area produced a complex flow configuration, as shown in Figure 13. The primary vortex (Vortex V1) is the largest and fastest moving anti-clockwise vortex that is closest to the apex. The created angular velocity in this instance induced the shed of the injected particles from the core vortex in the main vessel.

The primary vortex’s instability caused by the incoming flow from the bifurcation main vessel induced more fluctuations of the vortex in terms of the vortex dimension and vortex center position. This fluctuation is also influenced by the bifurcation angle. The influence of the bifurcation angle on the flow distribution and the particle deposition is not investigated in the present work. 

**Figure 14:**
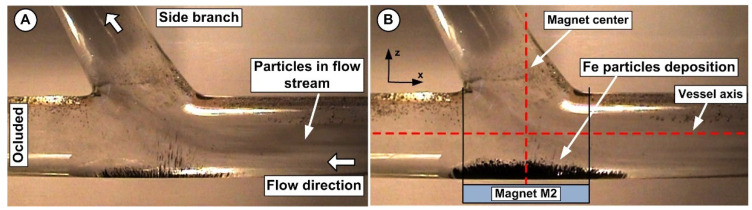
Fe particles deposit under the magnetic force generated by the permanent ferrite magnet having a grade of Y35 (magnet M2). Fe particles were injected for a period of 30 s. (**A**) Particle depositions over 5 s. (**B**) Depositions of particles at the end of the 30 s injection period. The magnet’s center in the z direction is in line with the vertical axis of the vessel bifurcation. The deposition shape shows how the drag force balances the magnetic force. Both investigations were conducted for identical working conditions, fluid velocity *V_f_* = 0.12 m/s, flow rate *Q* = 362 mL/min, Reynolds number of Re = 281, and distance between the vessel bottom wall and the magnet surface h = 5 mm.

Moreover, the flow division in the apex vicinity (Figure 14) boosted the interaction between the magnetic field, injected Fe particles, and fluid flow. As a result, some of the injected particles are transported downstream in the host vessel and its branches, and some of the particles get captured in the targeted areas. Additionally, the recirculation flow’s presence increases the particle near-wall residence time and facilitates the particle deposition around the bifurcation targeted region.

To present the influence of the flow rate on the particle depositions, we investigated experimentally two different flow regimes corresponding to the blood flow regime in the human arterial system [47] (Table 9).

Additionally, the results presented in Figure 14 demonstrated that ferrite-type magnets might be employed to deliver magnetoresponsive particles to a target location, even if they are less powerful than neodymium magnets.

All measurements regarding the vortex parameters and particle deposition characteristics (in terms of the length and thickness) were obtained using ImageJ (https://imagej.nih.gov/ij/ (accessed on 6 October 2022)).

It is important to note that the shape and number of Fe particles deposited in the desired area are affected by the viscous and magnetic forces described in Equations (3) and (4), respectively. The shape of the accumulating particles also provides information about how these forces were correlated during the injection phase.

### 5.2. The Effect of Magnet Type on Particle Accumulation

During these studies, the experimental measurements were performed three times for each investigated magnet position in the same way to show how well and consistently the magnetic targeting procedure worked.

In our case, the quantity of the Fe particle deposition m_Fe_ was determined experimentally from the weight difference between the injected amount of Fe particles in the suspension (m_Fe_total_ = 1 g) and the amount of the accumulated Fe particles in the targeted area. The Fe particles that had built up in the target area were collected after stopping the flow system and taking the permanent magnet out of the target area.

**Figure 15:**
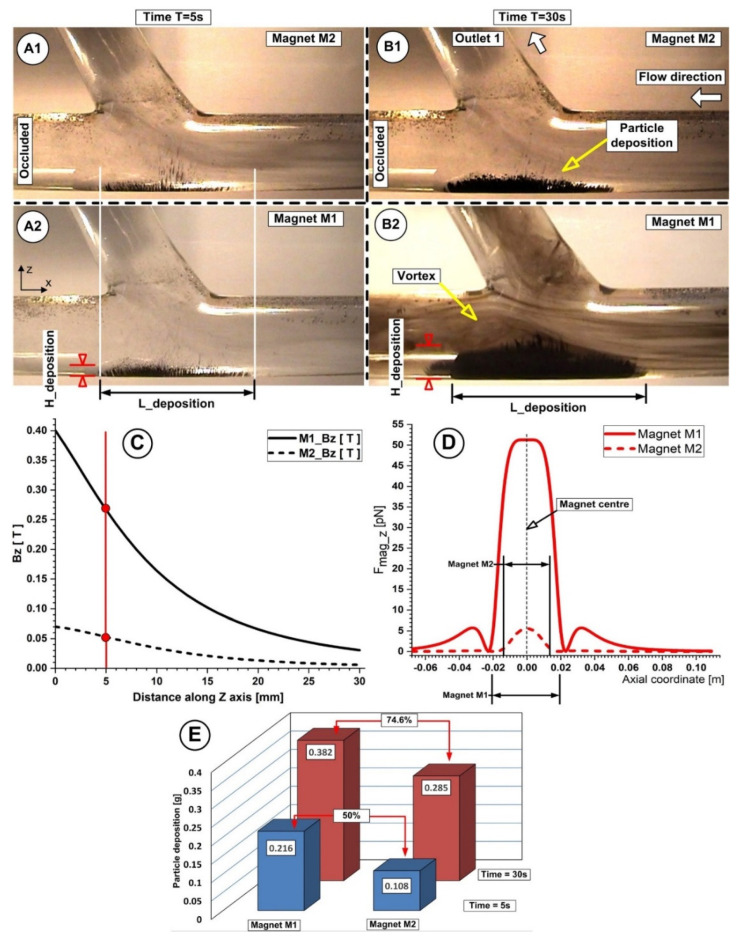
Particle retention dependency of the used magnet types (**A**,**B**). Particle accumulation evolution at different time steps during the injection period **A1**, **A2**, **B1**, **B2**. Magnetic field vertical component values for both magnets at h = 5 mm from the bottom wall of the artery (**C**). Magnetic force distribution for magnets M1 and M2 along the main artery wall (**D**). Comparison of the accumulated particle quantities and percentage differences between the used magnets, corresponding to the different time steps during injection (**E**). Investigations were conducted in the same conditions; distance from the wall of h = 5 mm, injection period of 30 s, flow rate of 362 mL/min, and Reynolds number Re = 281.

The quantitative correlations between the distance from the artery lower wall, magnetic field induction, and particle deposition quantities and shapes for different investigated magnet positions are presented in Table 10 and Figure 15. The measurements were performed for identical working conditions, namely the carrier fluid velocity v_f_ = 0.12 m/s, flow rate Q = 362 mL/min, Reynolds number of Re = 281, and an injection period of T = 30 s.

For all the investigated scenarios, the outlet Section 2 (Figure 5) was occluded. This investigated situation corresponds to the real anatomical situation in which the bifurcating artery is completely blocked off due to the main artery being completely clogged.

The captured Fe particles by the magnetic field generated by the used permanent magnets have a very distinct distribution throughout the host artery wall (as can be seen in Figure 15). The flow structure (recirculation position and length) (Figure 15(A1,A2,B1,B2)), flow velocity, and magnetic field intensity and field distribution (Figure 15C,D) are all closely related to the particle accumulation shape (Figure 15E) (length, thickness, and position) obtained at the end of the injection period (Table 10).

We specify two geometric parameters for the deposition of particles, namely the deposition length (L_deposition) and the deposition height (H_deposition), to examine the impact of the magnet utilized. The magnet’s size and the axial component of the magnetic force are connected to the length of the particle deposition. The vertical part of the magnetic force and primarily the type of magnet employed are related to the height of the deposit. The magnetic field that each magnet produces (which depends on how strongly it is magnetized and shaped) depends on the type of magnet used.

The defined parameters or particle deposition (L_deposition and H_deposition) can be used in biomedical applications to determine how much medication to provide during treatment and to determine the area of applicability (the diameter of the targeted artery to avoid artery blockage or flow reduction induced by the particle accumulation in the targeted site) for the specific magnet type. The two specified parameters are connected to the hemodynamic force produced by the flow and the strength of the magnetic field generated by the type of magnet used, as mentioned previously.

Equations (13)–(15) can be used to calculate the relationship between the magnetic and hydrodynamic forces by adding the effects of the axial and vertical components of the flow velocity and the magnetic force to the particle velocity (Equation (12)).

We define the targeting efficiency (*TE*) in the targeted area (the lower wall of the artery bifurcation region) as the ratio between the amount of *Fe* particles accumulated in the targeted region (m_Fe_) and the total amount of the injected Fe particles m_Fe_total_ during an injection period of 30 s (Table 11). This definition highlights the differences between the investigated magnets from the perspective of the particle targeting technique (Equation (16)).

(16) $$TE=\frac{m_{Fe}}{m_{Fe\_total}}\times 100,$$

The analysis of the results of the deposition efficiency presented in Table 11 allows the formulation of the following conclusions:

The targeting efficiency for the magnet M2 (but not for the M1) depends non-monotonically on the magnet distance. We consider that the monotonic dependence of the magnet M1 is peculiar to our case. Because in this article we used ordinary magnets, the number of deposited particles is the result of the simultaneous action of several aspects, such as the geometric shape of the magnet, its magnetization, the position of the magnetic center of the magnet, etc. In the following steps, we intend to use more types of magnets, so we analyze the correlation between the degree of deposition efficiency and the distance of the magnet from the target area.

### 5.3. Correlation between Magnet Distance and Particle Deposition

**Figure 16:**
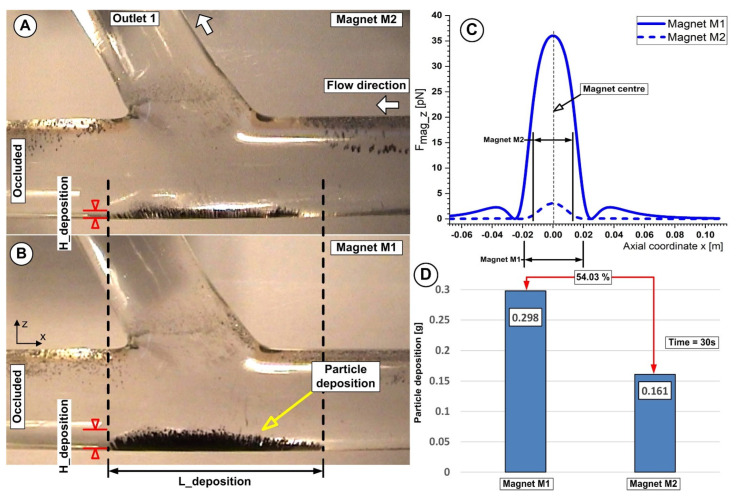
Particle accumulation dependency of the magnet distance h = 10 mm for both investigated magnets. (**A**) Magnet M2 is a ferrite magnet, and (**B**) Magnet M1 is a neodymium magnet. (**C**) Vertical magnetic force component for magnets M1 and M2 at a distance of h = 10 mm from the artery wall. (**D**) Accumulated Fe particle mass and mass percentage differences between magnets M1 and M2 at the end of the injection period. Working conditions were identical for both investigations; injection period of 30 s, the flow rate of 362 mL/min, and Reynolds number of Re = 281.

Figure 16 shows evidence of the correlation between the particle deposition and the magnet position relative to the artery bifurcation lower wall. As can be seen, the different magnet type induces differences both in the particle deposition length and the shape.

Additionally, from Figure 15A,B and Figure 16A,B we can see that Fe particles accumulate along the artery lower wall in a low-lying “dunes” shape.

Furthermore, the induced deposition by both types of magnets is divided into two distinct regions: a thinner region where the fluid flow washes away some of the collected Fe particles and a thicker region where the main recirculation zone (Vortex V1) and the generated magnetic field work together to catch the Fe particles.

Figure 16A,B and, respectively, Figure 15(B2) demonstrate how the build-up of particles alters the structure of the fluid flow in the target area by accelerating the fluid on the surface of the particle deposition, decreasing the flow section, and changing the size and location of the primary recirculation (vortex V1).

For the investigated magnets, we can state that the maximum deposition is associated with a minimum distance between the magnet and the target area. Furthermore, in the case of both magnets, a consistent deposition of particles is obtained for the distance of 10 mm from the magnet surface (26.8% for the magnet M1 and 16.1% for the magnet M2, respectively) (Figure 16D). From our point of view, it is essential to mention that this efficiency refers only to the fact that a reasonable number of particles are captured from the total injected. This effectiveness needs to be interpreted by medical experts to devise a plan for obtaining the best deposition from the point of view of the intended treatment.

Choosing the ideal distance for the magnet’s location is challenging. In the scenario examined in the present paper, the objective was to emphasize how the particle deposition can vary based on the type of magnet utilized and the location of the magnets in the targeted region (both in terms of the amount and deposition form). The “optimal distance” for the investigation described in the article refers to a distance between 5 and 10 mm. Considering the accumulated quantities of the Fe particle and the deposition shape, the optimal distance is 7 mm. This value is from the standpoint of using the drug-targeting process on the peripheral artery system, where the magnet’s (skin) surface and the distance between the peripheral arteries lie within this range. Once more, it is crucial to discuss these findings with the medical therapists to determine the “optimal distance” for each application.

## 6. Conclusions

A numerical simulation and a direct experimental measurement of the magnetic induction on the magnet system’s surface were used to map the magnetic field. The capacity of ferrite-type and neodymium permanent magnets to produce powerful magnetic forces was also examined analytically and quantitatively. Ansys-Maxwell software and FEMM 4.2 were used for all numerical computations in the current investigation. For both magnets, the generated magnetic fields were comparatively studied for targeting Fe particles having a diameter of 6 μm.

The following conclusions were drawn from a combined experimental and theoretical investigation of magnetic force production for targeted drug delivery applications:

(1) The deposition on the vessel wall is greatly influenced by the intensity of the magnetic field, the magnet type, the magnet size, and the magnetic characteristics of the ferromagnetic particles.
(2) How well particles may be targeted depends on how the magnetic and drag forces are balanced. This is because the magnetic and the drag forces are proportional to the particle’s size in terms of its cube.
(3) The results from the CFD models are qualitatively comparable with the measured magnetic field induction, magnetic field strength, and their fluctuation with the distance from the magnet surface.

In our opinion, the present article introduces some novel approaches for targeting applications, as follows:

(1) The used magnets are clearly defined and, more importantly, investigated from the magnetic field point of view.
(2) As described in Chapter 3.6, we used a glycerol-water solution during our experimental investigation to ensure that the rheological behavior of blood was replicated. There is a fundamental difference, to quantify the magnetic particle deposition in the in vitro test section, which tries to mimic the real working environment.
(3) Our investigation consists of a comparison between two different software and analytical solutions, respectively, which is a novelty in terms of results validation.
(4) In our research, we used ordinary commercially available magnets rather than special magnets or a magnet configuration designed for specific applications. Using ordinary magnets, we highlighted the possibility of creating an efficient magnetic system for an optimized drug-targeting technique.

The investigation of the efficiency of the deposition of magnetic particles in the presence of a magnetic field constitutes indisputable proof of the mode of action of the magnetic field on the particles injected into the fluid stream. This analysis of the deposition of particles from the point of view of the quantity of captured particles, the length of the deposit, and the shape of the deposition surface are the parameters that define the efficiency of the controlled deposition process and are directly correlated with the physical and magnetic properties of the magnets used. The number of captured particles are connected with the magnet’s type and degree of magnetization. The length of the deposition is correlated with the geometric shape of the magnet, the degree of magnetization, and the spatial position of the magnet. The shape of the deposition surface (keeping in mind that we have a circular section) is correlated with the flow rate of the working fluid, the pipe diameter, the roughness of the pipe walls, the magnet type, the intensity of the generated magnetic field, and the spatial position of the magnet.

It is feasible to analyze how factors (such as the magnet configuration, flow velocity, particle surface features, distance from the magnetic pole, and particle size) affect the magnetic targeting effectiveness to measure MP retention. Understanding the kinetics of how particles are collected and accumulated in magnetic drug-targeting applications can significantly impact the design and functionality of magnetic assemblies.

By comparing the findings of the numerical simulations performed under identical working conditions as in the case of actual investigations, the accuracy of the results achieved in the present work (magnetic particle deposition) will be evaluated for each imposed working condition. Therefore, it is necessary to implement numerical flow simulations in the presence of a magnetic field at the following stage of investigation.

## 7. Limitation

Vessel diameter, vessel shape (straight vessel, bifurcation, bypass graft, stenosed artery, curved artery, stented artery, etc.), flow rate, fluid viscosity, magnetic particle diameter and shape, and the amount of the magnetic particle injected are just a few of the variables that affect magnetic particle deposition. As a result, to accomplish the most significant particle deposition in the targeted location, each drug-targeting application requires a unique sort of strategy about the applied magnetic field (strength and spatial distribution).

The following factors contribute to the study’s limitations:

(1) The use of relatively large Fe particles (4–6 μm).
(2) The use of two different magnets (one neodymium and one ferrite) without being able to quantify the impact of each magnet’s geometry variation and level of magnetization on the effectiveness of capturing magnetizable particles.
(3) The large diameter of the test section (8 mm tubes).
(4) The vessel wall rugosity.
(5) The use of a single-flow regime.
(6) A single value for the artery bifurcation angle.

## 8. Outlooks

Better magnet systems are required going ahead for most therapeutic applications. More research is needed to figure out how and why the movement of magnetic nanoparticles gets better when a magnetic field is created.

Thus, in the first stage of the next study, we will focus on the effect of the flow on the deposition efficiency (a different patient-specific flow regime), and in the second step, we will investigate the deposition efficiency for magnets having the same geometries but different degrees of magnetization. These investigations aim to define an optimal magnetic–fluid flow combination that facilitates a controlled deposition of magnetic particles in the targeted area.

In the present studies, Fe particles were merely used to simulate the applicability of the magnetic drug-targeting approach for better drug carriers.

So, in our next studies, we plan to use both experiments and computer simulations with the spherical PEG-coated iron oxide nanocomposite.

## Figures and Tables

**Table 1:** Characteristics of the Fe particle.

Characteristics	Value
particle diameter	4–6 μm
density	7.86 g/cm^3^
molar mass	55.8 g/mol

**Table 2:** Magnetic properties of the Fe particle.

Saturation Magnetization	Saturation Field	Coercive Field	Remanent Magnetization
Ms (A·m^2^/kg): 177	Hs (kA/m): 600	Hc (kA/m): 1.32	Mr (A·m^2^/kg): 0.891

**Table 3:** Permanent magnets dimensions.

Magnet	Shape	Material	Length—L (mm)	Width—W (mm)	Thickness—T (mm)
M1	Rectangular	Neodymium—NdFeB	40	20	10
M2	Rectangular	Ferrite	26	29	5

**Table 4:** Permanent magnet properties.

Magnet	Shape	Magnetization Direction	Material	Grade	Br (T)	Hcb (kA/m)	Hcj (kA/m)	BHmax (kJ/m^3^)
M1	block	thickness	Neodymium	N52	1.42–1.47	860–995	≥955	380–422
M2	block	thickness	Ferrite	Y35	0.43–0.45	215–239	217–241	33.1–38.3

Where (BH)max, the maximum energy product; Br, remanence of magnetic flux density; Hcj, coercivity of magnetic polarization; and Hcb, coercivity of magnetic flux density.

**Table 5:** B-field values at a different position from the magnet surface and the percentage differences between analytical values and values obtained experimentally and by numerical analysis for neodymium N52-type permanent magnet.

Z-Position (mm)	Bz_Equation (T)	Bz_exp (T)	Bz_FEMM (T)	Bz_Maxwell (T)	(%) Diff. Equation—Exp	(%) Diff. Equation—FEMM	(%) Diff. Equation—Maxwell
0.00	0.4090	0.367	0.3532	0.3566	10.27	13.65	12.81
5.00	0.2727	0.247	0.2343	0.2371	9.42	14.08	13.05
10.00	0.1675	0.151	0.1456	0.148	9.85	13.09	11.64
15.00	0.1041	0.094	0.0923	0.0961	9.70	11.31	7.68
20.00	0.0671	0.061	0.0628	0.0663	9.09	6.43	1.19
25.00	0.0450	0.041	0.0443	0.0482	8.89	1.54	7.11
30.00	0.0312	0.026	0.0330	0.0367	16.67	5.80	17.63

z-position is presented in Figure 6A, corresponding to line L1. % diff. equation—exp = % difference between values measured experimentally and values from the theoretical model.

**Table 6:** Ferrite Y35-type permanent magnet B-field values compare different distances from the magnet surface.

Z-Position (mm)	Bz_Equation (T)	Bz_Exp (T)	Bz_FEMM (T)	Bz_Maxwell (T)	(%) Diff. Equation—Exp	(%) Diff. Equation—FEMM	(%) Diff. Equation—Maxwell
0.00	0.0780	0.0732	0.0476	0.0504	6.15	39.02	35.38
5.00	0.0589	0.0551	0.0371	0.04	6.45	37.00	32.09
10.00	0.0379	0.0351	0.0251	0.0281	7.39	33.71	25.86
15.00	0.0234	0.0215	0.0165	0.0194	8.12	29.40	17.09
20.00	0.0146	0.0131	0.0110	0.0138	10.27	24.98	5.48
25.00	0.0095	0.0085	0.0074	0.0101	10.53	22.32	6.32
30.00	0.0064	0.0058	0.0050	0.0077	9.38	21.64	20.31

z-position is presented in Figure 7A, corresponding to line L1.

**Table 7:** Magnetic suspension component properties.

Materials	Properties	Value	Unit
Carrier fluid	ρ_f_—fluid density η_f_—fluid dynamic viscosity χ_f_—fluid magnetic susceptibility	1055 0.0036 −6.6 × 10^−7^	kg/m^3^ kg/(m.s) [-]
Fe	ρ_Fe_—Fe particle density χ_Fe_—Fe particle magnetic susceptibility	7860 4	kg/m^3^ [-]

**Table 8:** Parameters used for magnetic and drag force calculation.

Symbol	Description	Default Value	Unit
m_eff_	Mass of the Fe particle (one particle)	8.88945 × 10^−13^	(kg)
$v_{p}$	Magnetic particle total velocity	$v_{p}=v_{f}+v_{mag}$	(m/s)
$v_{f}$	Fluid velocity (carrier fluid)	0.12	(m/s)
$v_{mag}$	Magnetic particle (Fe particle) velocity due to the magnetic force acting on the particle	$F_{mag}=\frac{1}{2}\mu_{0}\chi V_{p}\nabla H^{2}$	(m/s)
$v_{m}$	Fluid mean velocity (carrier fluid)	$V_{m}=\frac{Q}{S}=\frac{Q}{\pi R^{2}}$	(m/s)
$V_{p}$	The volume of magnetic particle	V_p_ = $\frac{4}{3}\pi R_{p}^{3}$	(m^3^)
$R_{p}$	Magnetic particle radius (Fe particle)	4 ÷ 6 × 10^−6^	(m)
R	Artery (vessel) radius	8 × 10^−3^	(m)
z	Magnetic particle (Fe) coordinates along the z direction		(m)
μ_0_	The magnetic permeability of air	μ_0_ = 4π × 10^−7^	(N/A^2^)
ρ_f_	Fluid density (carrier fluid)	1055	(kg/m^3^)
Q	Fluid flow rate (carrier fluid)	6.0288 × 10^−6^	(m^3^/s)
g	Gravity acceleration	9.81	(m/s^−2^)

**Table 9:** Flow regimes and primary vortex properties.

Item	Flow Rate Q (ml/min)	Flow Velocity v_f_ (m/s)	Reynolds Number Re (-)	Main Vortex Length L_V1 (mm)	Particle Deposition * (g)	Magnet Type	Magnet Distance h (mm)
Q1	362	0.12	281	17	0.285 ± 0.00616	M2	5
Q2	754	0.25	586	22	0.214 ± 0.00734	M2	5

* Measurements were performed three times for each investigated flow rate.

**Table 10:** Characteristics of the particle accumulation shape along the artery lower wall at the end of the injection period.

Magnet Distance h (mm)	Magnet Type	Magnetic Flux Density Bz (T)	Particle Deposition Quantity m_Fe_ (g)	Particle Deposition Length L_Deposition (mm)	Particle Deposition AVERAGE Thickness H_Deposition (mm)
2	M1	0.35	0.405 ± 0.00714	44	3
	M2	0.065	0.261 ± 0.00831	36	1.85
5	M1	0.27	0.382 ± 0.00817	41.5	2.2
	M2	0.051	0.285 ± 0.00616	34	1.11
10	M1	0.16	0.268 ± 0.00778	40	1.2
	M2	0.032	0.161 ± 0.00857	32	0.75

**Table 11:** Particle targeting efficiency (TE) in the artery bifurcation model.

Magnet Distance h (mm)	Magnet Type	Targeting Efficiency TE (%)	Targeting Efficiency Percentage Difference between M1 and M2
2	M1	40.5%	14.4%
	M2	26.1%	
5	M1	38.2%	9.7%
	M2	28.5%	
10	M1	26.8%	10.7%
	M2	16.1%	
